# Effects of an H3R Antagonist on the Animal Model of Autism Induced by Prenatal Exposure to Valproic Acid

**DOI:** 10.1371/journal.pone.0116363

**Published:** 2015-01-05

**Authors:** Diego Baronio, Kamila Castro, Taylor Gonchoroski, Gabriela Mueller de Melo, Gustavo Della Flora Nunes, Victorio Bambini-Junior, Carmem Gottfried, Rudimar Riesgo

**Affiliations:** 1 Translational Research Group in Autism Spectrum Disorders, Universidade Federal do Rio Grande do Sul, Porto Alegre, Brazil; 2 Postgraduate Program in Child and Adolescent Health, Universidade Federal do Rio Grande do Sul, Porto Alegre, Brazil; 3 Research Group in Neuroglial Plasticity, Department of Biochemistry, Universidade Federal do Rio Grande do Sul, Porto Alegre, Brazil; 4 Child Neurology Unit, Hospital de Clínicas de Porto Alegre, Universidade Federal do Rio Grande do Sul, Porto Alegre, Brazil; Harvard Medical School, UNITED STATES

## Abstract

Autism spectrum disorders (ASD) are a group of neurodevelopmental disorders primarily characterized by impaired social interaction and communication, and by restricted repetitive behaviors and interests. Ligands of histamine receptor 3 (H3R) are considered potential therapeutic agents for the treatment of different brain disorders and cognitive impairments. Considering this, the aim of the present study is to evaluate the actions of ciproxifan (CPX), an H3R antagonist, on the animal model of autism induced by prenatal exposure to valproic acid (VPA). Swiss mice were prenatally exposed to VPA on embryonic day 11 and assessed for social behavior, nociceptive threshold and repetitive behavior at 50 days of life. The treatment with CPX (3 mg/kg) or saline was administered 30 minutes before each behavioral test. The VPA group presented lower sociability index compared to VPA animals that were treated with CPX. Compared to the Control group, VPA animals presented a significantly higher nociceptive threshold, and treatment with CPX was not able to modify this parameter. In the marble burying test, the number of marbles buried by VPA animals was consistent with markedly repetitive behavior. VPA animals that received CPX buried a reduced amount of marbles. In summary, we report that an acute dose of CPX is able to attenuate sociability deficits and stereotypies present in the VPA model of autism. Our findings have the potential to help the investigations of both the molecular underpinnings of ASD and of possible treatments to ameliorate the ASD symptomatology, although more research is still necessary to corroborate and expand this initial data.

## Introduction

Autism spectrum disorders (ASD) are a group of neurodevelopmental disorders featured by impaired social interaction and communication, and by restricted repetitive behaviors and interests [1]. The pathophysiology of ASD is poorly understood, but evidence indicates that a strong genetic component and the environment act in concert as triggering factors [2,3].

The use of valproic acid (VPA) during pregnancy is an environmental risk factor highly associated with increased incidence of ASD in children [4]. Based on this observation, an animal model of this disorder was proposed, which consists of prenatally exposing rodents to this teratogen [5,6]. The VPA animal model of autism shows neuroanatomical, behavioral, and biochemical alterations that replicate the core characteristics and main comorbidities of patients with ASD [7].

Histamine acts as a transmitter in the central nervous system (CNS) and modulates distinct physiological processes like circulatory functions, innate and acquired immunity, cell proliferation and hematopoiesis [8]. In the last years, there has been a growing interest in the study of histamine in the CNS and its influence on behavior in both physiological conditions and brain disorders [9–11]. Thus, the histaminergic system is an interesting pharmacological target for therapeutic purposes and many efforts have been made to develop drugs that could act on different histamine receptors (H1R, H2R, H3R and H4R) [12].

There are few studies in the literature related to the use of histamine receptor antagonists to treat autistic behavior. In 1997, it was proposed that famotidine, an antagonist of H2R, would be a potential treatment for children with ASD [13]. This proposal was based on a report of a patient with schizophrenia (SCH), a disorder that shares symptoms and genetic factors with ASD [14,15], that showed improved sociability after this treatment [16]. Later, famotidine was tested in a group of children with ASD and 44% of them presented evidence of behavioral improvement. It is noteworthy that children with marked stereotypy did not respond to the treatment [17]. In addition, niaprazine, a H1R antagonist, was also tested in patients with ASD and led to amelioration of symptoms such as unstable attention, resistance to change and frustration [18].

Ligands of H3R are also considered potential therapeutic agents for the treatment of brain disorders, such as Alzheimer’s disease, SCH, and narcolepsy [19–21]. In an animal model of SCH, the use of an H3R antagonist ameliorates behavioral impairments [22], including spatial working memory deficit, an abnormality also found in patients with ASD [23]. Recently, a study revealed that antagonism of H3R attenuates impaired social behavior in rodents exposed to phencyclidine (PCP), a finding that may also have implications for ASD [24].

Considering this evidence, we aimed to evaluate the actions of ciproxifan (CPX), an H3R antagonist, on the animal model of autism induced by prenatal exposure to VPA. Sociability and social novelty preference, nociceptive threshold and repetitive behavior were assessed to verify the behavioral outcomes triggered by CPX treatment.

## Methods

### Animals

Thirty-six female Swiss mice were obtained at 7–8 weeks of age from Federal University of Pelotas (Pelotas, Brazil) and were mated, with pregnancy confirmed by the presence of a vaginal plug on embryonic day 0 (E0). On E11, 18 pregnant females received a single intraperitoneal injection of 500 mg/kg VPA (Sigma-Aldrich, St. Louis, MO, USA) dissolved in saline. Eighteen control females received an equal volume of saline. Four females died after injection of VPA. Sixteen pups from VPA-treated dams (VPA) and 16 pups from dams that received saline (Control) were used in this study. Half of each group received an injection of CPX prior to each assay, while the rest received an injection of saline (SAL). One pup was randomly selected from each litter in order to avoid litter effects. The other animals from the litters that were not used in this work were killed and tissues were stored for future analysis. Animals were maintained in a standard 12-hour light/dark cycle, with controlled temperature (22±2°C) and free access to food and water.

Behavioral testing was performed between 9:00 am and 2:00 pm, in an order randomized by group, in the following sequence once the animals were 50 days old: three chambers test, tail-flick, and marble burying. All protocols were approved by the Animal Ethics Committee at the Clinical Hospital of Porto Alegre (HCPA) and were conducted in accordance with National Institutes of Health guidelines.

### Treatment

CPX (Sigma-Aldrich, St. Louis, MO, USA) was dissolved in sterile 0.9% saline before the behavioral tests. Intraperitoneal injections of SAL or CPX (3 mg/kg) were given 30 minutes before onset of behavioral tests. Dosage was based on previous publications demonstrating efficacy in mice [25].

### Behavioral tests

#### Three Chambers test

This sociability and social novelty test apparatus consists of an acrylic box with a total size of 628 × 456 × 220 (length, width, height, in millimeters) partitioned into three chambers. The openings between the compartments allow the animals to explore the three chambers. During an initial moment, an object was positioned in one of the lateral chambers, and a set animal+object was placed in the opposite lateral chamber. This animal (novel mouse 1) was an experimentally naive male Swiss mouse with no previous contact with the testing animal. The object was an empty cage identical to the one used to enclose the novel mouse 1. Time spent in each chamber, as well as the time spent exploring the novel mouse 1 or the novel object, was analyzed by two observers during 10 min.

The social novelty test began immediately after the end of the sociability test. In this test, the novel mouse 1 remained in its wire cage (now it is called the known mouse) and a new unfamiliar mouse (novel mouse 2) was placed in the wire cage in the opposite side (which was previously empty). Time spent in each chamber and time spent exploring each wire cage was recorded during 10 min.

We evaluated a Sociability Index (SI), a mathematical equation designed to allow the direct comparison of social behavior of the groups. In an analogous manner, it is possible to evaluate preference for social novelty by calculating the Social Novelty Preference Index (SNI). The SI and SNI are calculated as showed below:

$$\frac{SI=(\mathrm{time exploring novel mouse}\;1-\mathrm{time exploring novel object})}{\left(\mathrm{time exploring novel mouse}\;1+\mathrm{time exploring novel object}\right)}$$

$$\frac{SNI=(\mathrm{time exploring novel mouse}\;2-\mathrm{time exploring known mouse})}{\left(\mathrm{time exploring novel mouse}\;2+\mathrm{time exploring known mouse}\right)}$$

#### Tail-flick test

Animals were gently restrained by hand, and radiant heat was directed onto its tail. Measurements were taken three times, with 30-second intervals, using a tail-flick analgesia meter (EFF 300L-Light, Insight, Brazil).

#### Marble burying test

Mice were acclimated individually in a cage filled with 4 cm of fresh bedding for 10 minutes. To minimize neophobia and novelty-induced anxiety, mice had been previously exposed to the testing room during the three chambers test and tail-flick test. Following acclimation, mice were removed from the cage, and 20 black marbles were placed equidistant in a 4×5 arrangement. Mice were then returned to the same cage for 10 minutes. Following the 10-minute testing period, two observers counted the number of marbles that were more than 50% covered with bedding. After each testing period, the marbles were cleaned with 70% ethanol.

### Statistics

For the SI and SNI, marble burying and tail-flick tests statistical significance was assessed with a group (Control, VPA) × drug (SAL, CPX) ANOVA. Bonferroni post-tests were used to determine difference between individual groups.

The values measured for the animals in the three chambers test were integrated in a multivariate linear model to predict the impact of the treatment in the behavioral outcome. We used the Generalized Estimation Equations (GEE) in order to enable the comparison between multiple interdependent variables and overcome the necessity of normality and homoscedasticity. Two distinct analyses were performed, one for time in chambers and another for the interaction time. Group and chamber were considered independent variables and their influence over the dependent variable time was determined. Bonferroni post-test was used as the final evaluation.

All analyses were performed using the SPSS program, Version 20.0 (SPSS, Chicago, IL). The *p* values less than 0.05 were considered as statistically signiﬁcant.

## Results

### Three chambers test

#### Sociability

**Figure 1:**
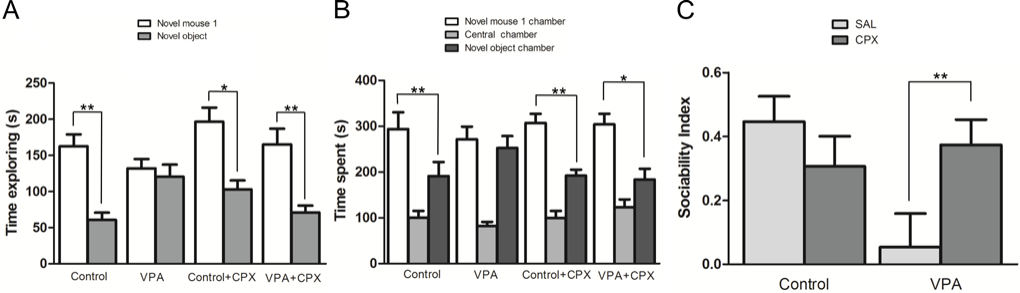
Sociability assessed by the three chambers test. After 5 minutes of acclimatization, male subjects were allowed to explore all chambers for 10 min. With the results obtained in the sociability assessment, the sociability index was calculated as the ratio of the difference to the sum of time spent exploring the novel mouse 1 and the time spent exploring the novel object. **(A)** Time spent exploring novel mouse 1 or novel object. **(B)** Time spent in chambers. **(C)** Sociability Index. Figures show mean ± SEM. (*p<0.05, **p<0.01). Control (n = 8), VPA (n = 8), Control +CPX (n = 8), VPA+CPX (n = 8).

The Control mice spent significantly more time exploring the novel mouse 1 than the novel object (p<0.01). In contrast, VPA mice showed no preference for the two stimuli, which could reflect decreased sociability. Interestingly, VPA animals treated with CPX spent significantly more time exploring the novel mouse 1 than the novel object (p<0.01) (Fig. 1A).

With exception of VPA animals treated with CPX, all the other animals spent less time in the central chamber than in the other chambers (p<0.01). The VPA mice treated with CPX spent significantly less time in the central chamber when compared to the novel mouse 1 chamber (p<0.01), but no difference was detected when the time spent in the central chamber was compared to the time spent in the novel object chamber (p>0.05). Control animals that received SAL and CPX spent significantly more time in the novel mouse 1 than in the novel object chamber (SAL: p<0.01; CPX: p<0.01), while the time spent by the VPA group in either chamber was not significantly different. After CPX treatment, VPA-exposed animals spent significantly more time in the novel mouse 1 chamber than in the novel object chamber (p<0.05) (Fig. 1B).

Regarding the SI, there was a significant group by drug interaction (F (1, 28) = 6.55, p = 0.016). No significant difference was detected between VPA and Control groups (F (1, 28) = 3.25, p = 0.082). However, when the SI of VPA animals that received only the SAL was compared to Control animals in the same conditions, a significant difference was detected (p<0.01, Bonferroni post-test). The SI of VPA mice treated with CPX was significantly higher than the SI of VPA animals that received only SAL (p<0.05, Bonferroni post-test). Treatment with CPX had no effect on Control animals (Fig. 1C).

#### Social novelty preference

**Figure 2:**
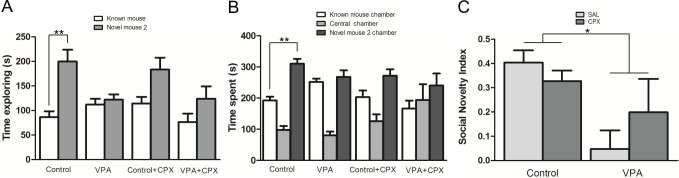
Social Novelty Preference assessed by the three chambers test. Right after the sociability assessment, male subjects were allowed to explore all chambers for 10 min. With the results obtained in the social novelty assessment, the social novelty index was calculated as the ratio of the difference to the sum of time spent exploring the novel mouse 2 and the time spent exploring the known mouse. **(A)** Time spent exploring known mouse or novel mouse 2 **(B)** Time spent in chambers. **(C)** Social Novelty Index. Figures show mean ± SEM. (*p<0.05, **p<0.01). Control (n = 8), VPA (n = 8), Control +CPX (n = 8), VPA+CPX (n = 8).

Control mice spent significantly more time exploring the novel mouse 2 than the known mouse (p<0.01). In contrast, VPA animals spent similar time exploring the two stimuli. Unlike what happened in the sociability assessment, Control and VPA animals treated with CPX spent similar time exploring the known and novel mouse 2 (Fig. 2A).

**Figure 3:**
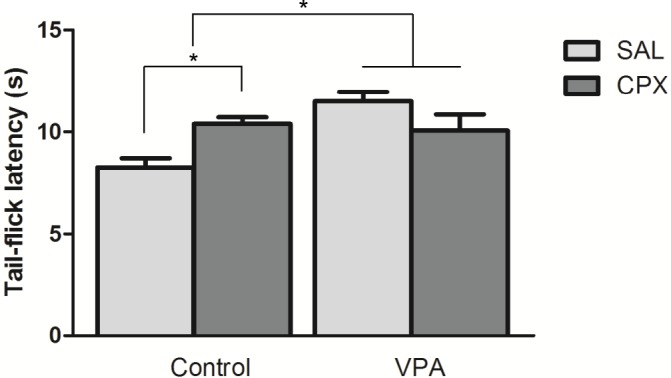
The effect of H3R antagonist CPX on thermal nociceptive threshold. Animals were restrained by hand and the nociceptive threshold was measured three times with intervals of 30 s, in order to calculate mean values. Figure shows mean ± SEM. (*p<0.05). Control (n = 8), VPA (n = 8), Control +CPX (n = 8), VPA+CPX (n = 8).

Control and VPA animals spent significantly less time in the central chamber than in the other chambers (p<0.01). Control mice treated with CPX spent less time in the central chamber than in the novel mouse 2 chamber (p<0.01), but it was not statistically different from the time spent in the known mouse chamber. The time spent by VPA-exposed mice treated with CPX in the central chamber was not statistically different from the time spent in the other two chambers. The Control group spent significantly more time exploring in the novel mouse 2 than in the known mouse chamber (p<0.01). As expected, the time spent in both chambers by VPA mice did not differ statistically. Interestingly, CPX had no effect on the time spent by Control mice in the chamber with novel mouse 2 and in the one with the known mouse (Fig. 3B). In the SNI, Control mice presented a higher score than VPA animals (F (1, 28) = 6.01, p = 0.021). There was no significant group by drug interaction (F (1, 28) = 2.20, p = 0.148). Treatment with CPX had no effect on VPA-exposed animals (p<0.05, Bonferroni post-test) (Fig. 2C).

### Tail-flick test

Animals from the VPA group presented a higher nociceptive threshold than the Control group (F (1, 28) = 7.25, p = 0.012). There was a significant group by drug interaction (F (1, 28) = 11.19, p = 0.002). Treatment with CPX enhanced the nociceptive threshold of Control mice (p<0.05, Bonferroni post-test). No effect was detected in VPA animals after treatment with CPX (Fig. 3).

### Marble Burying test

**Figure 4:**
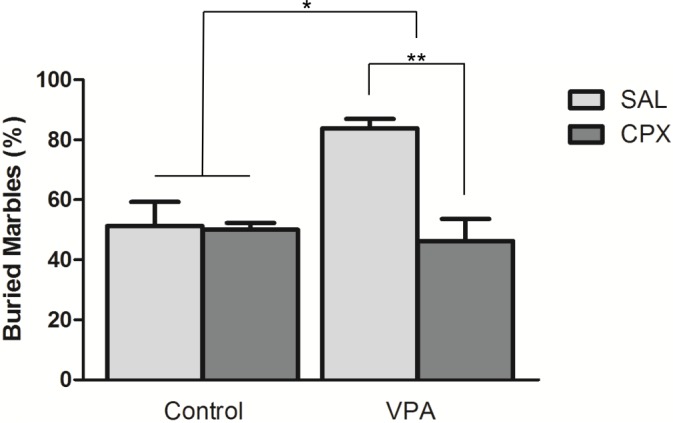
The H3R antagonist CPX attenuates elevated repetitive behavior in mice exposed to VPA in utero. Repetitive marble burying behavior was measured after a 10 minute testing session. VPA mice demonstrated elevated stereotyped, repetitive behaviors that were significantly reduced by CPX. Figure shows mean ± SEM. (*p<0.05, **p<0.01). Control (n = 8), VPA (n = 8), Control +CPX (n = 8), VPA+CPX (n = 8).

We assessed the effect of CPX on marble burying activity (Fig. 4). The VPA group buried significantly more marbles than the Control (F (1, 28) = 6.12, p = 0.020), consistent with an increase in repetitive behavior. There was a significant group by drug interaction (F (1, 28) = 9.73, p = 0.004). Marble burying was significantly reduced in VPA-exposed mice treated with CPX compared to VPA animals that received only SAL (p<0.01, Bonferroni post-test). However, no difference was detected between Control animals treated with SAL and treated with CPX.

## Discussion

The H3R antagonists are considered promising alternative treatments for different brain disorders, such as SCH, Alzheimer’s disease and narcolepsy [19]. The present study investigated, for the first time, the effects of the H3R antagonist CPX on a mouse model of autism based on prenatal exposure to VPA. We demonstrated CPX efficacy in attenuating impaired social behavior and stereotypies in VPA mice.

In the three chambers test, sociability is the propensity to spend time exploring an unfamiliar animal, as compared to time spent exploring an object. Treatment with CPX normalized the impaired sociability displayed by animals from VPA model, since these animals presented a SI similar to Control group. Most of the studies about H3R antagonist and social behavior are focused on social memory [26], a parameter that is also altered in ASD [27], and this experiment is the first one to assess the effects on sociability. The mechanism by which this improvement was acquired is not clear, but it might be involved with the capacity of H3R antagonists to mediate the release of different neurotransmitters besides histamine, such as dopamine, serotonin and acetylcholine, in specific brain areas [28]. Assessing the levels of different neurotransmitters in brain structures of the VPA model, as well as in VPA animals treated with H3R antagonists, would help to understand which neural circuits could be involved in this behavioral improvement.

No effect of CPX was detected in the social novelty assessment. That was unexpected, since CPX is able to improve object recognition in chronically stressed rats, abolishing the memory deficits [29]. In an opposite manner, it was demonstrated that when H3R is activated by the agonist imetit, consolidation of object recognition memory is impaired [30]. In addition, H3R antagonist treated rats, of an animal model of SCH induced by PCP exposure, tend to pursue social novelty. In this particular study, control rats presented a novelty discrimination index (ratio of the time spent investigating an unfamiliar subject divided by the time spent investigating the familiar one) 3.5 times greater than adult rats neonatally pretreated with PCP. Treatment with SAR110894, a H3R antagonist, dose-dependently normalized this altered behavior [24].

The difference between Griebel et al. results and ours, regarding social novelty, could be explained by different reasons. *1*) the discordance in the method used to evaluate the preference for social novelty (Griebel et al. used a sequential presentation of known and novel animal, instead of the three chambers test); *2*) the differences in rodent species (Griebel et al. tested rats); *3*) differences between the actions of SAR110894 and CPX at the given doses and *4*) divergence between the physiological mechanisms underlying the social novelty discrimination deficits in these two models. In this regard, it is interesting to note that the social novelty discrimination impairments in VPA animals could be mediated by factors other than cognitive deficits. For example, VPA-exposed animals seem to present olfactory deficits at postnatal day 10 that could impair olfactory learning, which is important for social recognition [31].

The fact that histamine reduces nociceptive transmission when injected into the brain has been known for some time [32]. This was a reason to believe that enhanced histamine release mediated by H3R antagonists could lead to an analgesic effect. Factors like the dose utilized and how the drug is delivered play a determinant role in the efficacy of H3R antagonists on modulating nociceptive threshold. Central application of H3R antagonist thioperamide increased nociceptive threshold in a partial nerve-ligation model, while systemic application reduced it [33]. In addition, low systemic doses (1–5mg/kg) of thioperamide were not able to produce an analgesic effect in mice on the hot plate test [34,35]. On the other hand, higher doses (5–30 mg/kg) of the same drug were able to increase the nociceptive threshold [36,37].

Recently, CPX and pitolisant, another H3R antagonist, were tested in mice to evaluate thermal nociceptive thresholds. A high dose of pitolisant increased the thermal pain threshold, while CPX (10 mg/kg) produced no effect. The authors suggested that this effect of pitolisant on heat responses was independent of H3R [38]. In the present study, we tested a lower dose of CPX (3 mg/kg), and different effects were detected on the tail-flick test in Control and VPA groups. Contradictory results are found in the literature about pain sensitivity and ASD, therefore, it is a consensus that not all children with ASD react the same way to pain. Generally, the VPA model of autism shows less sensitivity to pain [6], similar to what was found in this study.

The widespread distribution of H3R on fibers throughout the brain and spinal cord, mediating different physiological processes, and the different structures and pharmacokinetic properties of the H3R antagonists, might explain the contradictory findings regarding H3R antagonism and nociception.

In the 80’s, the use of L-histidine, a precursor of brain histamine, modified the methamphetamine (MET)-induced stereotypy in mice [39]. Later, it was verified that treatment with an inhibitor of histamine synthesis, α-fluoromethylhistidine, would cause a contrary effect, enhancing stereotyped behavior [40]. Recently, a likely involvement of the histaminergic system in the pathophysiology of Tourette syndrome, a condition common among patients with ASD and featured by stereotypies, has been hypothesized. A premature termination codon (W317X) in the L-histidine decarboxylase (HDC) gene, the rate-limiting enzyme in histamine biosynthesis, was detected in patients with this syndrome, implying that diminished histaminergic neurotransmission could be related to the outcomes of this syndrome [41].

In the present study, VPA mice treated with CPX displayed a reduced repetitive behavior in the marble burying test. Stereotypy and behavior rigidity are widely known as core and defining features of ASD. There are contradictory reports about the efficacy of H3R antagonists on suppressing these impairments. It was reported that CPX (3 mg/kg) was able to suppress locomotor sensitization induced in mice by MET. In addition, sensitization by MET also led to a decrease of N-methyl-D-aspartate (NMDA)-receptor subunit 1 (NR1) mRNA in the cerebral cortex, hippocampus and striatum. The treatment with CPX restored the normal levels of NR1 mRNA [42]. Interestingly, knockout of NMDA receptors, including NR1, in parvalbumin interneurons generates autistic-like phenotypes [43]. On the other hand, no improvements or even exacerbation of hyperactivity were reported after treatment with H3R antagonists in stereotypies of rats [44].

Since it is clear that VPA negatively affects the glial and neuronal development in this model [45,46], and CPX was administered just prior to the behavioral testing, it is unlike that we detected a total reversal in the autistic-like behaviors. At this time point, many of the changes that occurred during the CNS development (such as the deficits in migration, proliferation and network-establishment) reached a state of functional equilibrium and theoretically could not be easily modified. Nevertheless, a single application of CPX is already enough to improve behavioral deficits. This fact supports the hypotheses that at least some of the main clinical alterations present in ASD could be attenuated even in a late time stage.

In summary, we report that an acute dose of CPX is able to attenuate at least some sociability deficits and stereotypies present in the animal model of autism induced by VPA. More research is still necessary to corroborate and expand this initial data, and to contribute to generate a better understanding of ASD pathophysiology and management.
